# Estimation of energy consumed by middle-aged recreational marathoners during a marathon using accelerometry-based devices

**DOI:** 10.1038/s41598-020-58492-8

**Published:** 2020-01-30

**Authors:** Carlos Hernando, Carla Hernando, Ignacio Martinez-Navarro, Eladio Collado-Boira, Nayara Panizo, Barbara Hernando

**Affiliations:** 1 Sport Service, Jaume I University, Castellon, Spain; 2 Department of Education and Specific Didactics, Jaume I University, Castellon, Spain; 3 Department of Mathematics, Carlos III University of Madrid, Madrid, Spain; 4 Department of Physical Education and Sport, University of Valencia, Valencia, Spain; 5 Sports Health Unit, Vithas-Nisa 9 de Octubre Hospital, Valencia, Spain; 6 Faculty of Health Sciences, Jaume I University, Castellon, Spain; 7 Department of Medicine, Jaume I University, Castellon, Spain

**Keywords:** Public health, Translational research

## Abstract

As long-distance races have substantially increased in popularity over the last few years, the improvement of training programs has become a matter of concern to runners, coaches and health professionals. Triaxial accelerometers have been proposed as a one of the most accurate tools to evaluate physical activity during free-living conditions. In this study, eighty-eight recreational marathon runners, aged 30–45 years, completed a marathon wearing a GENEActiv accelerometer on their non-dominant wrist. Energy consumed by each runner during the marathon was estimated based on both running speed and accelerometer output data, by applying the previously established GENEActiv cut-points for discriminating the six relative-intensity activity levels. Since accelerometry allowed to perform an individualized estimation of energy consumption, higher interpersonal differences in the number of calories consumed by a runner were observed after applying the accelerometry-based approach as compared to the speed-based method. Therefore, pacing analyses should include information of effort intensity distribution in order to adjust race pacing appropriately to achieve the marathon goal time. Several biomechanical and physiological parameters (maximum oxygen uptake, energy cost of running and running economy) were also inferred from accelerometer output data, which is of great value for coaches and doctors.

## Introduction

Running a marathon has rapidly become one of the most popular activities nowadays as shown by the number of amateur participants with hundreds of marathons worldwide^1,2^. It is well-known that running a marathon is one of the most challenging endurance competitions^3,4^. As a result of recent research focused on improving training programs, which aimed to avoid soreness and prevent energy deficit during ultraendurance races^5^, the number of runners crossing the finish (ultra)marathon line has significantly raised over the past few years^6,7^. For example, a total of 3,388 runners more finished the Valencia Fundación Trinidad Alfonso EDP Marathon in 2018 as compared to the 2016 edition (19,246 *versus* 15,858 finishers, respectively)^8^.

In their way towards the improvement of marathon time, recreational runners are surrounded by a wide range of professionals in order to achieve their objectives^9,10^. Consequently, many studies has been focused on developing different methodologies to evaluate factors affecting running performance, such as the pacing strategy^2,11^, the energy consumption^12–14^, the maximal oxygen uptake $(\dot{V}{{\rm{O}}}_{2{\rm{\max }}})$^15^, the fraction of $\dot{V}{{\rm{O}}}_{2{\rm{\max }}}$ maintained (F)^15^, the running speed^16^, the energy cost of running (Cr)^17^, and physical, biomechanical, metabolic, psychological and social factors^18^.

Among all these factors, changes of running speed over race sections have been widely studied in order to explain the running success of more efficient pacers – runners who are able to maintain their initial running pace for more kilometers^2^. These more efficient pacers may avoid an excessive energy consumption while running the first part of the marathon^5^.

Therefore, measuring the energy expended by an individual while performing a specific activity has recently been targeted by researchers. Ainsworth and colleagues published The Compendium of Physical Activities in 1993 (which was reviewed in 2000 and 2011), allowing to directly extrapolate the energy expenditure in Metabolic Equivalent Task (METs), and thus in kilocalories (kcal), for running activities according to speed^12,13,19^.

Since the Compendium did not take into account interpersonal differences, the use of accelerometry-based devices has been proposed to evaluate free-living physical activities performed by an individual, in terms of duration, frequency and intensity^14,20,21^. Therefore, using the cut-points recommended for a specific population and/or activity, accelerometer output data can be applied to indirectly measure the energy expended by an individual in METs^22–24^.

In this regard, our research group aimed to monitor middle-aged recreational marathoners during a marathon using accelerometry-based devices. For this purpose, we previously established the GENEActiv cut-points that dsicriminate the six relative-intensity activity levels in recreational marathoners^25^. This lab-based study was essential in order to delineate specific GENEActiv cut-points for a specific population who presents higher relative level of fitness than the standard adult population. At this point, the main goal of the current study was to apply the GENEActiv cut-points previously established for estimating the energy consumed by middle-aged recreational marathoners during a marathon race (a free-living condition). Accelerometer output data allowed us to analyze the effort distribution that runners followed to achieve their marathon time, by means of the time running at each one of the six related-intensity levels (sedentary, light, moderate, vigorous, very vigorous and extremely vigorous activity) during the marathon. This information may be extremely valuable for both athletes and coaches. Knowing the intensity, duration and energy cost of an activity is useful for designing training sessions because it allows to objectively quantify and monitor training load. Energy consumption was also estimated based on running speed^12^, and results were compared with those obtained after using accelerometer data.

## Results

A detailed description of individuals included in this study is summarized in Table 1.

**Table 1:** Population description.

Variable		Subjects (N = 88)
Physiological characteristics*	age	38.68 ± 3.61
	BMI	22.91 ± 1.62
	Weight	69.96 ± 8.91
	Heigh	174.44 ± 8.66
	% body fat	14.74 ± 4.38
	$\dot{V}{{\rm{O}}}_{2{\rm{\max }}}$ (ml·kg^−1^·min^−1^)	54.41 ± 5.66
	maximum METs	15.55 ± 1.62
Training indicators*	years of running	6.43 ± 2.78
	sessions per week	4.90 ± 0.84
	kilometers per week	63.45 ± 13.06
	hours per week	7.44 ± 2.70
History as marathoner*	marathons finished	3.36 ± 3.02
	marathon per year	1.10 ± 0.63
Work intensity^#^	high intensity	7.95%
	medium intensity	30.68%
	low intensity	61.36%
Levels of study^#^	school graduate	4.60%
	high school graduate	6.90%
	professional certificate	17.24%
	undergraduate degree	71.26%

Abbreviations: N, number of samples; BMI, body mass index; SD, standard deviation.

*Values are presented as mean ± SD.

^#^Values are presented as percentage.

The accelerometer output data allowed us to analyse the effort distribution that runners followed to achieve their marathon time, by means of the time running at each one of the six related-intensity levels (sedentary, light, moderate, vigorous, very vigorous and extremely vigorous activity) during the marathon. Values established for delineating the six-relative intensity levels of physical activity are detailed in Table 2.

**Table 2:** Values established for delineating the six-relative intensity levels of physical activity.

Relative-intensity levels of physical activity^#^	Reference values established for each intensity level by Hernando *et al*.^25^		Values used for energy consumption estimation		
	$\dot{{\bf{V}}}{{\bf{O}}}_{{\bf{2}}}$ (ml·kg^−1^·min^−1^)	METs*	${\boldsymbol{ \% }}\dot{{\bf{V}}}{{\bf{O}}}_{{\bf{2max}}}$	$\dot{{\bf{V}}}{{\bf{O}}}_{{\bf{2}}}$ (ml·kg^−1^·min^−1^)	METs*
Sedentary *X* < 10%	$\dot{V}{{\rm{O}}}_{2} < 5.45$	METs < 1.56	8.26%	4.5	1.29
Ligth 10% ≤ *X* < 25%	$5.45\le \dot{V}{{\rm{O}}}_{2} < 13.63$	1.56 ≤ METs < 3.90	17.5%	9.54	2.73
Moderate 25% ≤ *X* < 45%	$13.63\le \dot{V}{{\rm{O}}}_{2} < 24.54$	3.9 ≤ METs < 7.01	35.0%	19.10	5.45
Vigorous 45% ≤ *X* < 65%	$24.54\le \dot{V}{{\rm{O}}}_{2} < 35.44$	7.01 ≤ METs < 10.13	55.0%	29.99	8.57
Very Vigorous 65% ≤ *X* < 85%	$35.44\le \dot{V}{{\rm{O}}}_{2} < 46.35$	10.13 ≤ METs < 13.24	75.0%	40.90	11.69
Extremely Vigorous *X* ≥ 85%	$\dot{V}{{\rm{O}}}_{2}\ge 46.35$	METs_ ≥ _13.24	92.5%	50.44	14.41

Abbreviations: N, number of individuals; $\dot{V}{{\rm{O}}}_{2{\rm{\max }}}$, maximum oxygen consumption; $\dot{V}{{\rm{O}}}_{2}$_,_ oxygen consumption; MET, metabolic equivalent task.

Each minute of the cardiopulmonary test was classified into one of the six intensity categories of physical activity relative to an individual’s level of cardiorespiratory $(\dot{V}{{\rm{O}}}_{2{\rm{\max }}})$.

*1 MET = 3.5 ml·kg^−1^·min^−1^. 1 MET = 1 kcal·h^−1^.

^#^*X* denotes the percentage of a person’s aerobic capacity $(\dot{V}{{\rm{O}}}_{2{\rm{\max }}})$ used to classify each one of the six relative-intensity categories.

For all individuals, we estimated the energy cost of running a marathon, presenting the caloric consumption for each one of the 9 marathon sections as well as for the full marathon distance (Tables 3 and 4). The calories consumed by each runner were calculated based on both accelerometer data (Table 3), as previously described by our research group^25^, and running speed (Table 4), following the methodology proposed by Ainsworth and cols^12^. The aim of applying also the speed-based method^12^ in the estimation of energy consumption was to compare the results obtained with accelerometer devices^25^. Note that a gold standard method for energy quantification in long distance races has not been defined yet.

**Table 3:** Evaluation of effort distribution and estimation of calories consumed by runners based on accelerometry data.

Race section	Time spend at each relative-intensity level (minutes)							Energy consumed according to the time spend at each relative-intensity level (kcal)						
	S	L	M	V	VV	EV	Total	S	L	M	V	VV	EV	Total
0–5 km	0.01 ± 0.11	0.00 ± 0.00	1.17 ± 4.87	1.30 ± 4.03	9.82 ± 10.65	14.81 ± 11.53	27.10 ± 3.35	0.02 ± 0.15	0.00 ± 0.00	6.76 ± 26.60	13.94 ± 45.35	136.71 ± 148.23	244.83 ± 191.55	402.26 ± 76.44
5–10 km	0.00 ± 0.00	0.00 ± 0.00	1.42 ± 4.31	1.63 ± 4.00	8.67 ± 8.94	12.86 ± 10.32	24.58 ± 2.23	0.00 ± 0.00	0.00 ± 0.00	8.28 ± 23.95	17.16 ± 44.77	119.77 ± 123.75	214.47 ± 173.67	359.68 ± 73.47
10–15 km	0.00 ± 0.00	0.00 ± 0.00	1.25 ± 3.66	1.84 ± 4.30	8.56 ± 8.94	13.09 ± 10.24	24.74 ± 2.32	0.00 ± 0.00	0.00 ± 0.00	7.84 ± 20.59	19.07 ± 45.53	118.40 ± 126.80	216.93 ± 171.78	362.25 ± 69.49
15-HM	0.01 ± 0.11	0.00 ± 0.00	1.88 ± 4.90	2.23 ± 4.44	9.74 ± 10.07	16.16 ± 12.47	30.01 ± 2.87	0.01 ± 0.12	0.00 ± 0.00	11.62 ± 28.85	23.10 ± 48.98	135.17 ± 141.08	267.77 ± 208.66	437.67 ± 87.00
HM-25km	0.00 ± 0.00	0.01 ± 0.11	0.51 ± 2.09	1.23 ± 3.48	6.06 ± 7.41	11.72 ± 8.45	19.52 ± 1.77	0.00 ± 0.00	0.03 ± 0.29	3.02 ± 12.38	12.57 ± 37.79	84.05 ± 102.70	195.15 ± 143.59	294.83 ± 57.25
25–30 km	0.00 ± 0.00	0.01 ± 0.11	1.13 ± 2.94	1.91 ± 3.84	8.33 ± 8.41	14.11 ± 10.23	25.49 ± 2.51	0.00 ± 0.00	0.04 ± 0.33	6.85 ± 17.57	19.15 ± 38.97	115.57 ± 118.10	235.14 ± 172.56	376.75 ± 72.58
30–35 km	0.00 ± 0.00	0.06 ± 0.38	1.53 ± 4.75	1.81 ± 3.95	8.06 ± 8.74	15.06 ± 11.00	26.51 ± 3.45	0.00 ± 0.00	0.20 ± 1.40	10.00 ± 31.54	18.34 ± 40.36	110.92 ± 121.80	250.91 ± 186.13	390.38 ± 77.84
35–40 km	0.00 ± 0.00	0.09 ± 0.58	2.08 ± 5.38	1.64 ± 3.28	8.22 ± 8.66	15.11 ± 10.51	27.14 ± 3.89	0.00 ± 0.00	0.33 ± 2.21	13.50 ± 36.03	16.04 ± 31.78	114.47 ± 120.83	251.14 ± 175.75	395.48 ± 72.99
40-M	0.02 ± 0.21	0.02 ± 0.15	0.67 ± 2.22	0.39 ± 0.84	2.55 ± 3.30	6.24 ± 4.10	9.89 ± 1.76	0.03 ± 0.31	0.07 ± 0.47	4.23 ± 13.93	3.79 ± 8.19	35.79 ± 8.19	104.43 ± 70.04	148.35 ± 37.76
Marathon	0.05 ± 0.34	0.19 ± 0.92	11.6 ± 25.32	13.95 ± 27.75	69.99 ± 66.19	119.16 ± 82.86	214.98 ± 20.78	0.06 ± 0.47	0.67 ± 3.48	72.10 ± 160.10	143.17 ± 301.99	970.84 ± 938.15	1980.78 ± 1386.54	3167.63 ± 584.12

Abbreviations: S, Sedentary; L, Light; M, Moderate; V, Vigorous; VV, Very Vigorous; EV, Extremely Vigorous; HM, Half marathon; M, marathon; SD, standard deviation.

Values are presented as mean ± SD.

**Table 4:** Comparison between accelerometry- and speed-based approaches in the estimation of energy consumption.

Race section	Running speed (m·min^−1^)	Absolute energy (kcal)		Energy relative to body mass per time (kcal·kg^−1^·min^−1^)			Energy relative to body mass per distance (kcal·kg^−1^·km^−1^)			Number of BMR		
		Accelerometry	Running speed*	Accelerometry	Running speed*	Adjusted *p-*value^¥^	Accelerometry	Running speed*	Adjusted *p-*value^¥^	Accelerometry	Running speed*	Adjusted *p-*value^¥^
0–5 km	187.27 ± 23.06	402.26 ± 76.44	352.30 ± 44.85	0.214 ± 0.031	0.189 ± 0.023	**6.27 × 10**^**-12**^	1.154 ± 0.195	1.008 ± 0.026	**1.09 × 10**^**-11**^	12.82 ± 1.84	11.30 ± 1.40	**6.27 × 10**^**-12**^
5–10 km	205.06 ± 18.43	359.68 ± 73.47	354.24 ± 47.26	0.210 ± 0.034	0.208 ± 0.019	0.149	1.030 ± 0.176	1.012 ± 0.023	0.495	12.59 ± 2.03	12.43 ± 1.11	0.149
10–15 km	203.85 ± 18.88	362.25 ± 69.49	355.00 ± 46.26	0.211 ± 0.032	0.207 ± 0.018	0.062	1.040 ± 0.171	1.015 ± 0.025	0.169	12.63 ± 1.93	12.38 ± 1.10	0.062
15-HM	204.94 ± 18.82	437.67 ± 87.00	427.90 ± 54.89	0.210 ± 0.033	0.206 ± 0.020	0.093	1.030 ± 0.177	1.003 ± 0.020	0.358	12.57 ± 2.00	12.31 ± 1.17	0.088
HM-25km	201.49 ± 18.00	294.83 ± 57.25	273.44 ± 35.86	0.217 ± 0.030	0.202 ± 0.022	**1.05 × 10**^**-5**^	1.055 ± 0.164	1.001 ± 0.026	**2.46 × 10**^**-3**^	12.99 ± 1.78	12.10 ± 1.33	**1.05 × 10**^**-5**^
25–30 km	198.01 ± 19.10	376.75 ± 72.58	353.60 ± 47.10	0.213 ± 0.030	0.200 ± 0.020	**7.85 × 10**^**-5**^	1.080 ± 0.170	1.010 ± 0.024	**3.16 × 10**^**-4**^	12.73 ± 1.82	11.98 ± 1.21	**7.85 × 10**^**-5**^
30–35 km	191.43 ± 22.58	390.38 ± 77.84	351.74 ± 46.67	0.213 ± 0.032	0.193 ± 0.025	**2.06 × 10**^**-7**^	1.119 ± 0.186	1.006 ± 0.040	**8.18 × 10**^**-7**^	12.73 ± 1.89	11.55 ± 1.49	**2.06 × 10**^**-7**^
35–40 km	187.65 ± 24.50	395.48 ± 72.99	353.24 ± 46.98	0.211 ± 0.032	0.190 ± 0.026	**7.21 × 10**^**-7**^	1.134 ± 0.174	1.010 ± 0.039	**2.37 × 10**^**-10**^	12.65 ± 1.91	11.36 ± 1.56	**7.21 × 10**^**-7**^
40-M	229.14 ± 42.02	148.35 ± 37.76	153.73 ± 20.93	0.215 ± 0.034	0.229 ± 0.038	0.202	0.964 ± 0.210	1.000 ± 0.039	1.000	12.90 ± 2.03	13.69 ± 2.26	0.209
Marathon	198.06 ± 18.78	3167.63 ± 584.12	2951.45 ± 394.20	0.212 ± 0.030	0.198 ± 0.021	**3.48 × 10**^**-5**^	1.076 ± 0.163	0.999 ± 0.023	**8.75 × 10**^**-5**^	12.70 ± 1.77	11.86 ± 1.23	**3.48 × 10**^**-5**^

Abbreviations: BMR, Basal metabolic rate; HM, Half marathon; M, Marathon; SD, standard deviation; *p*, *p*-value.

Values are presented as mean ± SD.

Bold indicates significant results (*p*-value < 0.05).

*The values are estimated based on running speed, and following the methodology proposed by Ainsworth *et al*. (2000)^12^.

^¥^*P-*values were corrected for multiple comparisons by applying the Benjamini-Hochberg procedure for decreasing the False Discovery Rate.

Except for the last race section, a higher number of calories was estimated to be consumed by a runner when the accelerometry-based method was applied, as compared to the caloric consumption estimated by using the speed method (Table 4). It is worth highlighting that a greater variation of calories consumed per each individual was observed after using accelerometry for energy cost estimation, rather than running speed (shown by higher standard deviation values). The reason of this difference is due to the fact that the accelerometer-based method takes into account the variability across individuals in terms of energy consumption, while speed-based method tends to standardize values for all subjects^26^.

**Figure 1:**
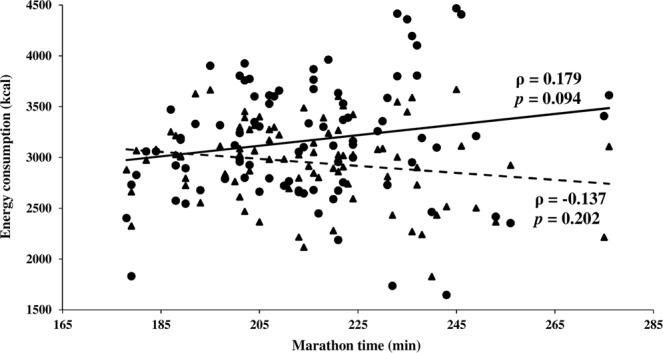
Plot showing the linear correlation between the calories estimated to be consumed by each runner and the marathon time. Energy consumption was estimated by using both accelerometry (solid line) and running speed (dashed line). Each individual is represented by a specific point: filled circles are used when accelerometry was applied for energy consumption estimation, and filled triangles when speed-based method was used. Abbreviations: ρ, Spearman’s rank correlation coefficient; *p*, *p*-value.

Although no significant differences between energy consumption and marathon time were observed (Fig. 1), correlation analysis showed that the accelerometry-based method tended to increase the number of calories consumed by the runner with marathon time (ρ = 0.179, *p* = 0.094). However, the Ainsworth’s method seemed to present a negative correlation between the caloric consumption and marathon time (Fig. 1). This correlation was also no significant (ρ = −0.137, *p* = 0.202).

For a better comparison between methods, the energy consumed by runners was expressed as a relative rate in kilocalories per kilogram of body mass either per minute^12,26^ or per kilometer^17,27^, and as the number of times consuming his/her Basal Metabolic Rate (BMR)^26,28^ (Table 4). The results of this comparison denoted statistically significant differences in the energy estimated to be consumed by runners after applying the accelerometry- and speed-based method. That was observed in each one of the 9 race sections as well as in the full marathon distance (Table 4).

Accelerometer output data allowed us to know the physical effort distribution of runners during the marathon, in terms of physical activity intensity. That is, we were able to identify and quantify when a runner is racing at each one of the six relative-intensity activity levels (sedentary, light, moderate, vigorous, very vigorous and extremely vigorous)^25^. Therefore, following the values established in Table 2, the percentage of $\dot{V}{{\rm{O}}}_{2{\rm{\max }}}$ produced per each runner was estimated, and this allowed then to calculate the energy of cost running above standing (Cr_net_)^28^ (Table 5).

**Table 5:** Estimation of the percentage of $\dot{V}{{\rm{O}}}_{2{\rm{\max }}}$, the oxygen uptake relative to body mass per minute and the energy cost of running above standing based on accelerometry data.

Race section	Percentage of maximum oxygen consumption $( \% \dot{V}{{\rm{O}}}_{2{\rm{\max }}})$	Oxygen uptake relative to body mass per minute (ml·kg^−1^·min^−1^)	Energy cost of running above standing* (J·kg^−1^·m^−1^)
0–5 km	82% ± 11.78	44.87 ± 6.43	4.54 ± 0.83
5–10 km	81% ± 13.05	44.07 ± 7.12	4.05 ± 0.76
10–15 km	81% ± 12.41	44.19 ± 6.77	4.09 ± 0.73
15-HM	81% ± 12.83	44.00 ± 7.00	4.04 ± 0.76
HM-25km	83% ± 11.46	45.45 ± 6.25	4.26 ± 0.72
25–30 km	82% ± 11.69	44.54 ± 6.38	4.25 ± 0.73
30–35 km	82% ± 12.11	44.55 ± 6.60	4.40 ± 0.79
35–40 km	81% ± 12.27	44.28 ± 6.70	4.44 ± 0.74
40-M	83% ± 13.06	45.15 ± 7.12	3.79 ± 0.86
Marathon	81% ± 11.38	44.43 ± 6.21	4.23 ± 0.70

Abbreviations: HM, Half marathon; M, Marathon; $\dot{V}{{\rm{O}}}_{2{\rm{\max }}}$, maximum oxygen consumption.

*Energy cost of running above standing = ($(\dot{V}{{\rm{O}}}_{2}-\dot{V}{{\rm{O}}}_{2{\rm{standing}}})$ (running speed)^−1^) · 20.9.

**Figure 2:**
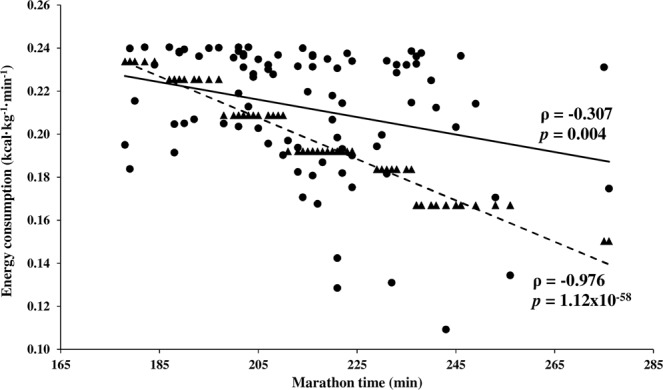
Plot showing the linear correlation between the energy estimated to be consumed by each runner relative to his/her body mass per minute and the marathon time. Energy consumption was estimated by using both accelerometry (solid line) and running speed (dashed line). Each individual is represented by a specific point: filled circles are used when accelerometry was applied for energy consumption estimation, and filled triangles when speed-based method was used. Abbreviations: ρ, Spearman’s rank correlation coefficient; *p*, *p*-value.

A negative correlation between the relative energy consumed and the marathon time was observed when energy consumption was expressed as kilocalories per kilogram of body mass per minute. This negative correlation was enlarged when the speed-based method was applied (ρ = −0.976, *p* = 1.12 × 10^−58^), in comparison with the accelerometry-based method (ρ = −0.307, *p* = 0.004) (Fig. 2). When the relative rate of energy consumption was expressed per distance (kcal·kg^−1^·km^−1^), the energy expended by runners was positively correlated with the marathon time after using accelerometry (ρ = 0.402, *p* = 1.01 × 10^−4^). No significant correlation was observed between energy consumption (expressed as a relative rate per kilogram of body weight per kilometre) and time when speed-based method was applied (ρ = −0.200, *p* = 0.062).

## Discussion

In this study, we aimed to estimate the energy consumed by middle-aged recreational marathoners during a marathon race (a free-living condition) using accelerometry-based devices^25^. In our opinion, the application of accelerometers should be useful to minimize the interpersonal differences in energy consumption caused by physiological and biomechanical parameters and, therefore, to perform an individualized estimation of energy consumption.

Up to now, the viability of accelerometers to measure $\dot{V}{{\rm{O}}}_{2}$ in combination with other devices, such as pulsometers or global positioning system (GPS) devices, has been analysed under laboratory conditions^29–31^. Accelerometers have also been used to monitor athletes and infer their physical activity level^24,32,33^. However, accelerometry-based devices had not been applied so far for estimating the energy consumed by a runner in a marathon race, under normal race conditions, yet. By applying the GENEActiv cut-points for discriminating the six relative-intensity activity levels in recreational marathoners (previously established in a lab-based study by our research group^25^), we were able to know the amount of time that a runner was running at a specific relative-intensity level (sedentary, light, moderate, vigorous, very vigorous and extremely vigorous activity) during the marathon. Accordingly, the energy consumed by the runner along the race sections and the full marathon distance was estimated.

Differences in the estimation of runners’ energy consumption were observed between the speed- and accelerometry-based methods. These differences lie in the ability of the accelerometer output data to determine the physical effort distribution of each runner during the marathon, in terms of physical activity intensity^34–36^. Therefore, accelerometers are able to perform an individualized estimation of energy consumption. Note that several physiological and biomechanical factors that are unique to the individual have been shown to affect the running efficiency among runners at the same steady-state speed^16,27,37^. This fact pointed up that estimating the energy consumption of a runner based uniquely on his/her running speed might be insufficient and that it might be advisable to apply a correction factor for adjusting for individual differences when estimating the energy cost of, at least, moderate/vigorous physical activities^26^. The speed-based approach, proposed by Ainsworth and cols^12^, analyse the marathon pace of a runner without taking into account the runner’s effort to race at this speed. Fewer interpersonal differences in the number of calories consumed by a runner were then observed with the speed-based method as compared to the accelerometry-based approach. For example, two individuals racing at identical speed and having equal body mass are estimated to present the same energy cost after applying the speed-based method, although their physical efforts are completely different according to accelerometry data. Nevertheless, note that, as in the speed-based methods, accelerometry is not able to perform an absolute quantification of the energy consumed by a runner and it is necessary, therefore, to combine different approaches, as well as to explore other technologies, in future work.

In this regard, accelerometer data collected for each runner was thoroughly analyzed in order to compare effort distribution between the fastest and the slowest runner of our dataset (Table 6). Note that the fastest runner was almost running at very vigorous intensity level, showing a good control of physical effort along the full marathon distance. In contrast, the effort distribution of the slowest runner was far from being well-balanced^2,38,39^. In fact, the accelerometer data revealed a considerable decay of the intensity level at which the slowest runner performed after completing 30 km (running at a moderate intensity from an extremely vigorous level). This was a consequence of the high physical effort sustained by the runner from the beginning of the marathon line, which reveals the importance of controlling effort distribution in a marathon race. In short, our results suggest that future pacing analyses should include information of effort intensity distribution in order to adjust race pacing appropriately to achieve the marathon goal time.

**Table 6:** Comparison of effort distribution according to accelerometer output data between the fastest and the slowest runner of our dataset.

Fastest runner: Marathon time of 178 min, body mass of 69.2 kg, and BMI of 21.36 kg·m^−2^													
Race section	Time running at each relative-intensity level (min)							Energy consumption			Running speed (m·min^−1^)	$\% \dot{V}{{\rm{O}}}_{{\rm{2max}}}$	Cr_net_ (J·kg^−1^·m^−1^)
	S	L	M	V	VV	EV	Total	Absolute (kcal)	Relative to time (kcal·kg^−1^ ·min^−1^)	Relative to distance (kcal·kg^−1^ ·km^−1^)			
0–5 km	0	0	0	0	21	0	21.00	283.70	0.20	0.82	238.10	75.00%	3.29
5–10 km	0	0	0	0	21	0	21.00	283.70	0.20	0.82	238.10	75.00%	3.29
10–15 km	0	0	0	1	20	0	21.00	280.09	0.19	0.81	238.10	74.05%	3.25
15-HM	0	0	0	3	22	0	25.00	326.92	0.19	0.77	243.90	72.60%	3.11
HM-25km	0	0	0	0	16	0	16.00	216.15	0.20	0.80	243.91	75.00%	3.22
25–30 km	0	0	0	0	22	0	22.00	297.21	0.20	0.86	227.27	75.00%	3.45
30–35 km	0	0	0	1	21	0	22.00	293.60	0.19	0.85	227.27	74.09%	3.41
35–40 km	0	0	0	0	18	3	21.00	293.13	0.20	0.85	238.10	77.50%	3.40
40-M	0	0	0	0	7	2	9.00	127.87	0.21	0.84	243.89	78.89%	3.38
Marathon	0	0	0	5	168	5	178.00	2402.37	0.20	0.82	237.05	74.93%	3.31
**Slowest runner: Marathon time of 276 min, body mass of 74.9 kg, and BMI of 23.38 kg·m**^**−2**^													
0–5	0	0	0	0	24	5	29.00	441.06	0.20	1.18	172.41	78.02%	4.73
5–10	0	0	0	0	17	11	28.00	446.85	0.21	1.19	178.57	81.88%	4.79
10–15	0	0	1	0	7	20	28.00	469.66	0.22	1.25	178.57	86.07%	5.04
15-HM	0	0	0	0	4	31	35.00	617.25	0.24	1.35	174.21	90.50%	5.43
HM-25	0	0	0	0	0	22	22.00	396.54	0.24	1.36	177.39	92.50%	5.45
25–30	0	0	2	4	13	12	31.00	462.89	0.20	1.24	161.29	76.61%	4.97
30–35	0	0	41	1	1	0	43.00	304.84	0.09	0.81	116.28	36.40%	3.27
35–40	0	0	43	0	1	0	44.00	307.75	0.09	0.82	113.64	35.91%	3.30
40-M	0	0	11	0	0	5	16.00	165.11	0.14	1.00	137.19	52.97%	4.04
Marathon	0	0	98	5	67	106	276.00	3611.95	0.17	1.14	152.88	67.16%	4.59

Abbreviations: S, Sedentary; L, Light; M, Moderate; V, Vigorous; VV, Very Vigorous; EV, Extremely Vigorous; HM, Half marathon; M, marathon; $\dot{V}{{\rm{O}}}_{2{\rm{\max }}}$, maximum oxygen consumption; Cr_net_, energy cost of running above standing.

Thanks to accelerometer output data, we were also able to estimate the percentage of $\dot{V}{{\rm{O}}}_{2{\rm{\max }}}$ produced per each runner, and afterwards the energy of cost running above standing (Cr_net_)^28^, at each of the 9 marathon sections as well as at the full marathon distance. These physiological parameters seem to explain up to 87% of the long distance race performance^27^. In addition, the accelerometry-based approach also allowed us to extrapolate the running economy of each runner, which is considered an important physiological measure for long distance runners^37,40^. It is thought that a variety of biomechanical characteristics are likely to contribute to having interpersonal differences in the running efficiency, such as the running technique, the elastic power of the muscle-tendon unit, or the amount of ground contact and vertical oscillation when running^41^.

As results shown, the fastest runner seemed to present a better efficiency of movement than that presented by the slowest runner. That is, the energy demanded for a given running velocity was lower by the fastest runner as compared to the slowest runner. In fact, the average energy cost of marathon running was 3.31 J·kg^−1^·m^−1^ for the fastest runner (whose average speed was 237.05 m·min^−1^), while it was 4.59 J·kg^−1^·m^−1^ for the slowest runner (whose average speed was 152.88 m·min^−1^). Apart from physiological parameters, these differences may be also resulted from biomechanical efficiency, which is influenced by anthropometric parameters, kinematic characteristics and running style^37^.

This suggests that the design of training sessions for the slowest runner by his coach should focus on improving his running style and muscle strength, and subsequently his performance. The useful information offered by accelerometers (distribution of physical effort in free-living conditions and inference of physiological parameters as Cr_net_ or % $\dot{V}{{\rm{O}}}_{2{\rm{\max }}}$) should become more and more important as race distance increase^42^. Application of accelerometers to monitor ultratrail runners may be useful not only for adjusting race strategy, which is crucial for achieving performance goals^2,27,43,44^, but also to monitor training sessions and recovery time. Indeed, both long-term data collection and wrist watch-like format are valuable characteristics of accelerometers since data can be continuously collected for a long period of time (more than a week) without causing any physical discomfort to ultraendurance runners^45^.

However, values of all physiological parameters analyzed in this study were merely estimations based on accelerometer data, and were not directly measured^46^. It is quite difficult, if not impossible, to perform a direct measurement of $\dot{V}{{\rm{O}}}_{2}$ on a marathon race, an extremely demanding free-living condition. This makes difficult to find a gold standard method for quantifying calories consumed by an individual when she/he is performing a physical activity. That is the reason why indirect measurement methods (such as heart-rate recording devices^14,47^, pedometers^48,49^ and accelerometers^14,34,36^, or their combination^29,30,50^) are normally applied. Another limitation of our study is related to the protocol followed to estimate energy consumption according to the range of % $\dot{V}{{\rm{O}}}_{2{\rm{\max }}}$ delimiting each relative-intensity activity level. Estimations can present a maximum error of 10%, since the median value of the % $\dot{V}{{\rm{O}}}_{2{\rm{\max }}}$ range was used for energy calculations (as shown in Table 2). Having said that, our results indicate that accelerometry-based method allows to both identify the individual’s levels of physical activity intensity during the marathon race and estimate an individualized energy consumption.

In summary, overall the results in this study lead us to believe that GENEActiv. accelerometer is an accurate tool for estimating the energy consumption of middle-recreational marathoners running a marathon, an extremely demanding free-living physical activity. Accelerometer-derived data was useful to evaluate the effort intensity distribution along the race, by means of the time running at each six related-intensity levels (sedentary, light, moderate, vigorous, very vigorous and extremely vigorous activity), and subsequently to estimate the energy consumption. Therefore, accelerometers may be extremely useful for both athletes and coaches who need to evaluate the race strategy to achieve marathon final time, but also to monitor training sessions and assess performance level progression needed to reach a goal. Several physiological and biomechanical parameters that can be inferred from accelerometer output data may also support coaches to design specific training sessions according to runner’s characteristics. Furthermore, the ability to perform an objective assessment of a runner’s fitness level, as well as energy consumption, in the context of free-living movement indicates that accelerometry-based devices may be of great value to sport medical professionals.

Since accelerometry-based data is thought to be valuable for monitoring runners along ultra-trail races, future studies determining cut-off points for quantifying energy consumption would help in the race strategy in terms of food and fluid intake on race day (a key factor for performance success). Note that these future studies must take into account that biomechanics and physiology of downhill and uphill running, as well as the energy cost of running, may differ.

## Methods

### Sample set

A total of 95 recreational marathon runners (80 males and 15 females) aged between 30 and 45 years lined up at the start of the Valencia Fundación Trinidad Alfonso EDP 2016 Marathon (20^th^ November, 2016). From all of them, eighty-eight participants crossed the finish line (74 males and 14 females). Non-finishers were discarded from further analyses. The entire process of sampling (contact approach and criteria for inclusion and exclusion of volunteers) has been previously described^25^.

### Ethics statement

All individuals included in the current study were fully informed and gave their written consent to participate. The research was conducted according to the Declaration of Helsinki, and it was approved by the Research Ethics Committee of the University Jaume I of Castellon. This study is enrolled in the ClinicalTrails.gov database, with the code number NCT03155633 (www.clinicaltrials.gov).

### Data collection and analysis

Four weeks before the marathon, we made an appointment with all participants in order to collect anthropometric data, demographics, medical information, training program and competition history. Indeed, all individuals completed a cardiopulmonary test. Details of data collection, processing and analysis have been previously described^25^. Population description according to data collected is also available in our previous work^25^.

All participants were weighed one hour before the start of the marathon, wearing racing clothes and flats, by using a Seca 770 scale (Seca Hamburg, Germany). BMI was then calculated (height·mass^−2^).

For this research, all the participants underwent the same testing under the same experimental conditions. Participants completed the Valencia Fundación Trinidad Alfonso EDP 2016 Marathon, which was held in November with a mean dry temperature of 15.6 °C and a mean relative humidity of 50%. The race course altitude varied from 1 to 27 m above sea level.

During the race, participants wore a GENEActiv accelerometer (Activinsights Ltd., Kimbolton, Cambridgeshire, United Kingdom). The accelerometer was worn on the non-dominant wrist as a watch. Accelerometers were adjusted to record acceleration data at a rate of 85.7 Hz. Devices were calibrated by the manufacturer prior to use. Processing of acceleration data has been previously explained in detail^25^.

### Data analysis

The marathon race was divided into 9 sections as follow: 6 sections of 5 km (0–5 km, 5–10 km, 10–15 km, 25–30 km, 30–35 km and 35–40 km), 1 section of 6.0975 km (15–21.0975 km), 1 section of 3.9025 km (21.0975–25 km) and 1 section of 2.195 km (40–42.195 km). All data analyses were performed for each one of the nine marathon sections and for the whole marathon distance. Statistical analyses were done using the IBM SPSS Statistics v.23 software, and *p*-values lower than 0.05 were considered as statistically significant. Supplementary information includes raw data used in this study.

Firstly, accelerometer-derived data was used to determine the distribution of exercise intensity of runners along the marathon with the aim to estimate the calories consumed per each runner. The intensity levels of physical activity were established following the cut-off points delineated by Hernando and cols^25^. For calculating the energy cost, we used the median value of the range of % $\dot{V}{{\rm{O}}}_{2{\rm{\max }}}$ delimiting each intensity category (Table 2), except for the sedentary category where the standing oxygen cost (4.5 mlO_2_·kg^−1^·min^−1^) was applied as reference value^28^. As unit of measurement, we considered that one MET is equal to 3.5ml O_2_·kg^−1^·min^−1^, and one MET is equal to one kcal·kg^−1^·h^−1^. These equivalencies were applied in accordance with the determinations proposed by Ainsworth and cols^12^, and taking into account that all volunteers included in the study reported similar BMI (between 22.17 and 23.44 kg·m^−2^) and, therefore, differences in the percentage of fatty component among participants were absence^26,46,51^.

Accelerometers were also used to estimate the percentage of $\dot{V}{{\rm{O}}}_{2{\rm{\max }}}$ produced per each runner. Briefly, the time racing at a specific intensity level was multiplied by its corresponding % $\dot{V}{{\rm{O}}}_{2{\rm{\max }}}$ (Table 2). A weighted average relative to the total time spent at each section, as well as at the full marathon distance, was then performed. Then, the VO_2net_ of each runner was calculated by subtracting the VO_2standing_ to the percentage of $\dot{V}{{\rm{O}}}_{2{\rm{\max }}}$ estimated^17,28^. Together with the running speed measured, the VO_2net_ was finally used to calculate the energy of cost running above standing (Cr_net_), following the methodology proposed by di Prampero and cols^17^.

Next, the average running speed was used to calculate the caloric consumption of runners, following the methodology proposed by Ainsworth and cols^12^. The split-times in minutes were recorded for each one of the marathon sections electronically, and the average running speed of all sections and the whole marathon distance was calculated. Then, the running speed was associated with a specific MET value, which can be directly used to calculate the number of calories consumed by a runner^12,19^.

Finally, the relative values of energy consumption estimated by the two models were compared. As the energy consumption depends on the person’s body mass, the energy cost of each runner is presented as: (i) the calories consumed per kilogram of body weight per minute (kcal·kg^−1^·min^−1^), in order to obtain the effort intensity;^12,19,26^ (ii) the calories consumed per kilogram of body weight per kilometer (kcal·kg^−1^·km^−1^), to infer the running efficiency of runners;^18,27^ and (iii) as the number of Basal Metabolic Rate (BMR) consumed, used as an indicator of the effort intensity degree above the basal metabolism^26,28^.

The Kolgomorov-Smirnov test was used for testing data normality. Since variables were not normally distributed, all statistical analyses were performed by applying non-parametric statistical tests. The Mann-Whitney U test was used to compare the energy consumption values estimated by using the accelerometer-derived data and the relative running speed. Then, *P-*values were corrected for multiple comparisons by applying the Benjamini-Hochberg procedure for decreasing the False Discovery Rate.The Sperman’s correlation test was applied to analyze linear association between two continuous variables.

## Supplementary information

Dataset 1.

## Data Availability

All data generated or analysed during this study are included in this published article (and its Supplementary Information File). Any other relevant data can be obtained from the corresponding author upon reasonable request.
